# The dawn of the liquid biopsy in the fight against cancer

**DOI:** 10.18632/oncotarget.23131

**Published:** 2017-12-08

**Authors:** Irma G. Domínguez-Vigil, Ana K. Moreno-Martínez, Julia Y. Wang, Michael H.A. Roehrl, Hugo A. Barrera-Saldaña

**Affiliations:** ^1^ Departamento de Bioquímica y Medicina Molecular, Facultad de Medicina de la Universidad Autónoma de Nuevo León, Monterrey, Nuevo León, México; ^2^ Genetics Laboratory, Vitagénesis, Monterrey, Nuevo León, México; ^3^ Curandis Laboratories, New York, NY, USA; ^4^ Department of Pathology, Memorial Sloan Kettering Cancer Center, New York, NY, USA; ^5^ TecSalud, Tecnológico de Monterrey, San Pedro Garza García, Nuevo León, México

**Keywords:** liquid biopsy, cfDNA, ctDNA, early detection, diagnostics

## Abstract

Cancer is a molecular disease associated with alterations in the genome, which, thanks to the highly improved sensitivity of mutation detection techniques, can be identified in cell-free DNA (cfDNA) circulating in blood, a method also called liquid biopsy. This is a non-invasive alternative to surgical biopsy and has the potential of revealing the molecular signature of tumors to aid in the individualization of treatments. In this review, we focus on cfDNA analysis, its advantages, and clinical applications employing genomic tools (NGS and dPCR) particularly in the field of oncology, and highlight its valuable contributions to early detection, prognosis, and prediction of treatment response.

## INTRODUCTION

The U.S. National Cancer Institute (NCI) defines liquid biopsy (LB) as “a test done on a sample of blood to look for cancer cells from a tumor that are circulating in the blood or for pieces of DNA from tumor cells that are in the blood” [1]. In this review, we will primarily focus on the second part of this definition, i.e., the detection of circulating DNA.

**Figure 1:**
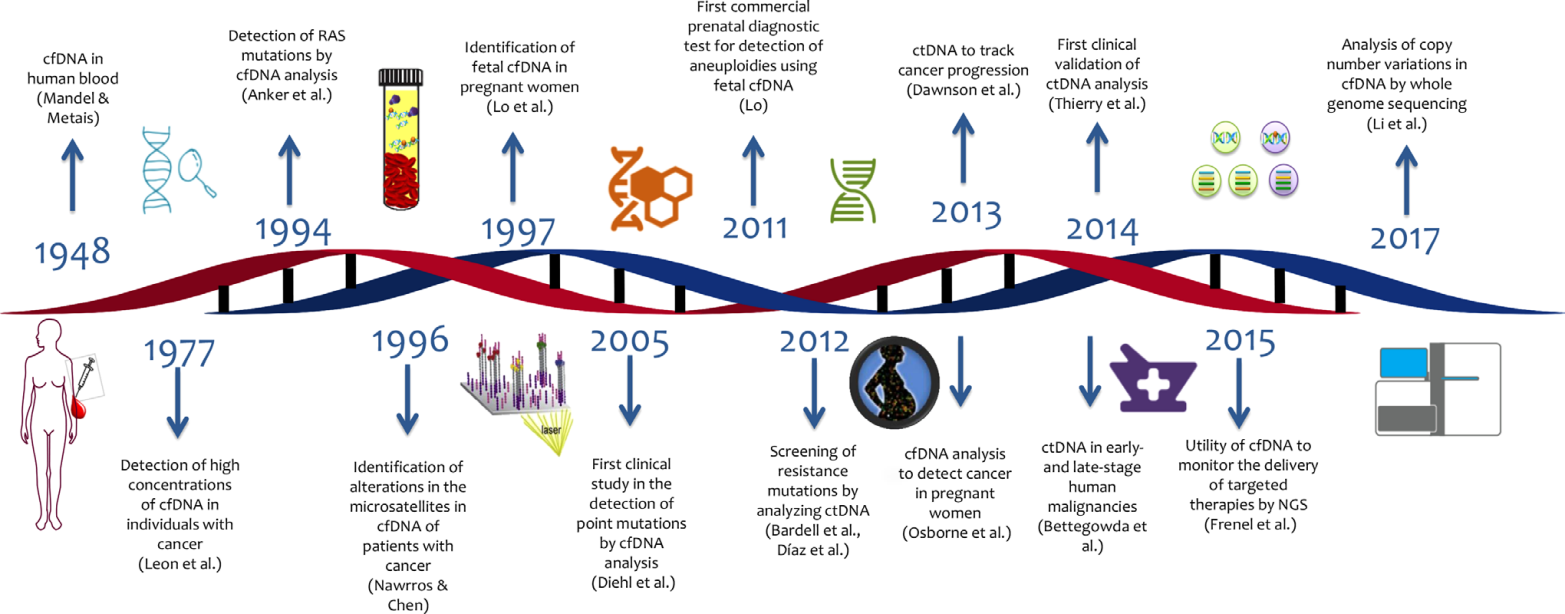
Timeline of liquid biopsy development

The first steps to explore the potential of cell free DNA for genetic testing of cancer were made in 1948, when the first publication of cell-free DNA (cfDNA) and free RNA circulating in human blood appeared [2]. Fast forwarding to today shows that numerous tests based on circulating nucleic acids are in development that – so is the hope – will help to opportunely discriminate patients with cancer from healthy individuals [3] (Figure 1).

**Figure 2:**
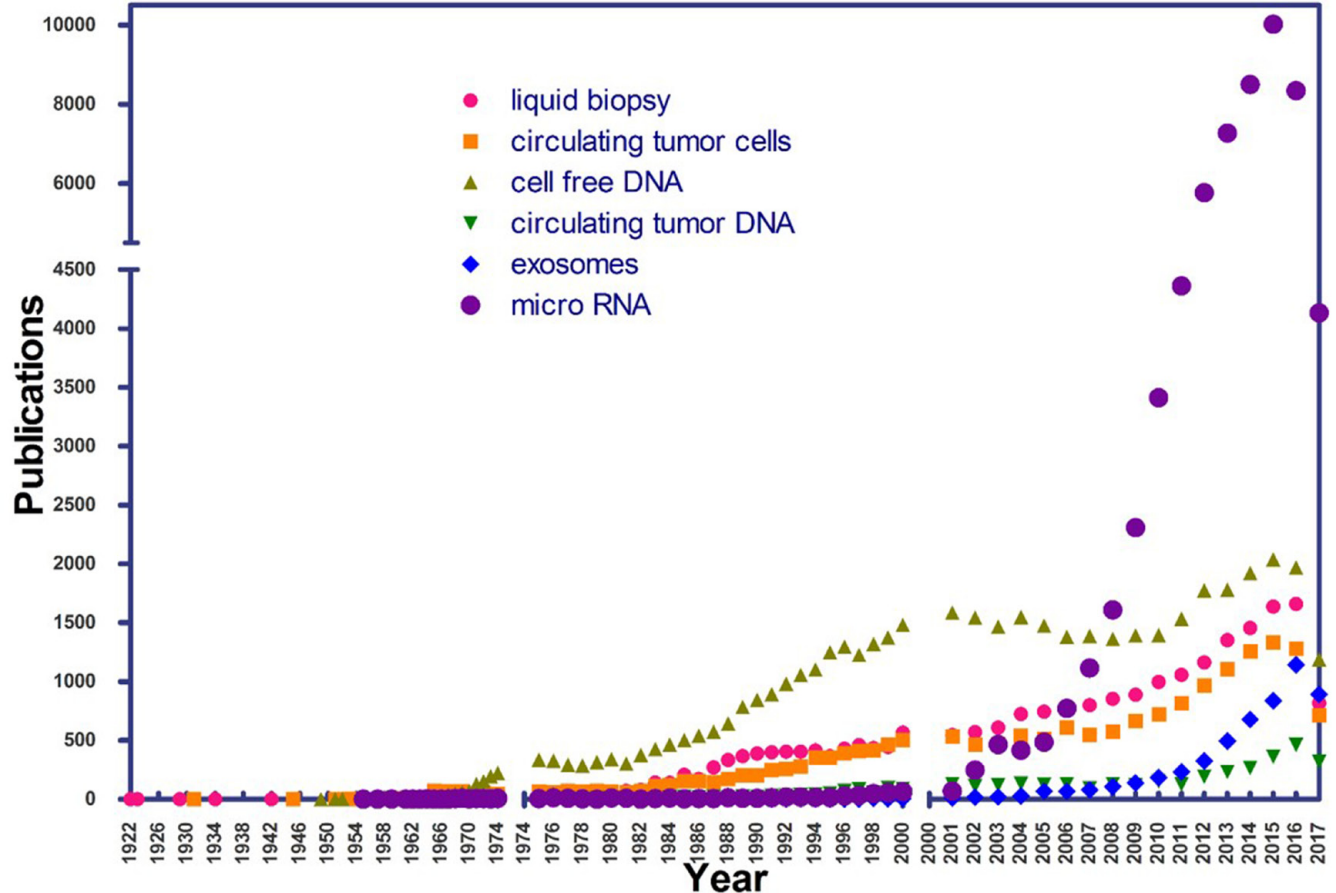
Number of publications per year in PubMed, using the terms “liquid biopsy”, “cell free DNA”, “circulating tumor DNA”, “exosomes”, “micro RNA”, and “circulating tumor cells” as of July 1, 2017

In its beginnings, the application of LB did not generate much attention on the part of the scientific community. A review of the PubMed (NCBI) database, using the search term “liquid biopsy” from 1975 onwards, shows a recent increase in the number of publications, denoting rapidly growing interest in LB (Figure 2).

### Rediscovering LB for diagnostic purposes

To the surprise of many, Osborne *et al*. reported in 2013 the case of a 37-year-old pregnant woman with a normal medical history but a non-invasive prenatal testing (NIPT) result suggestive of aneuploidy for chromosomes 18 and 13. NIPT searches for fetal cfDNA (3–13%) among the maternal cfDNA [4]. After spontaneous labor, a male fetus was born without dimorphic characteristics. The patient's vaginal biopsy revealed a small cell carcinoma with evidence of aneuploidy in 80% of analyzed cells, including alterations in chromosomes 18 and 13 that were consistent with the NIPT test performed previously. This was the first reported case of detection of cancer in a pregnant women by cfDNA [5].

### Genome alterations of cancer

Cancer is a pathological condition that encompasses more than 100 distinct disease entities with diverse risk factors and epidemiologic features and that can originate from essentially all cell types and organs of the human body. It is characterized by a relatively unrestrained proliferation of cells that can invade beyond normal tissue boundaries and metastasize to distant organs [6]. A hallmark of cancer is alterations in the genome. These alterations may be single nucleotide variants (SNVs), promoter methylation, copy number variation (CNVs), chromosomal structural rearrangements, and alterations in sites relevant for transcription, splicing, RNA maturation, or translational efficiency [7].

The International Cancer Genome Consortium (ICGC) and The Genomic Atlas of Cancer (TCGA), which aim to catalog the genomic information of the various types of cancer and foster discoveries that could allow better understanding of cancer origins and development [8], have carried out large-scale research into many different types of cancers in order to determine the genomic signatures of each. For example, mutations in the genes *ERBB2, PIK3R1, TP53,* and *NF1* stand out in glioblastoma [9]; *TP53, NF1, BRCA1, BRCA2, RB1, GABRA6, CSMD3, FAT3,* and *CDK12* alterations characterize ovarian cancer [10]; *TP53, PTEN, CTNNB1, PIK3CA, ARID1A, KRAS, ARID5B,* and *POLE* mutations are features of endometrial cancer [11]; *TP53, RAS, EGFR, BRAF, PIK3CA, MET, RIT1, STK11, KEAP1, NF1, RB1, CDKN2A, SETD2, ARID1A, SMARCA4, RBM10, U2AF1,* and *MGA* are hallmarks of lung cancer [12]. These studies were made possible with next generation sequencing (NGS) technology that has the advantage of simultaneously analyzing a large number of genes related to a specific phenotype, allowing the identification of mutations that are otherwise not easily detected [13]. One of the main advantages of NGS is that it allows global determination the molecular subtype of the disease (via large gene panel, exome, or even whole genome sequencing). It also makes monitoring progression of the disease and assigning targeted molecular therapies easier [8].

### Liquid biopsy in cancer

Cancer is often found in organs or tissues of the body that are difficult to access, such as brain, ovaries, or pancreas. Thus measurements of tissue-resident biomarkers for such cancers may be difficult or associated with significant clinical risk, such as bleeding or infection as consequence of an invasive biopsy or excisional procedure [14].

Surgical biopsies (SB) continue to dominate as the “gold standard” for diagnosis and choice of treatment for diseases of genetic and contagious origin. However, they also present disadvantages. Among them is the fact that tumor tissue sampling delivers only a static and spatially limited representation from the time of the surgical procedure. Cancers, however, vary over time due to continuous changes that result in genetic heterogeneity within the tumor and between the primary and metastatic sites (a characteristic typical of cancers in advanced stages). In addition, the majority of biopsies are commonly fixed in formalin and embedded in paraffin for routine pathology, which can reduce their utility for advanced molecular analyses [15]. Some of these disadvantages can be addressed with the implementation of LB.

LB holds great promise for detection, prognosis, and prediction of response to cancer treatment [16–18]. Among the main sources of LB-based biomarkers are circulating tumor DNA (ctDNA), circulating tumor cells (CTCs), exosomes, and microRNAs. ctDNA currently leads applications for diagnostic purposes, and, for this reason, it is the main subject of this review.

While we do not anticipate that LB will fully replace or directly compete with SB for most diagnostic purposes any time soon, we do very much foresee that LB will complement SB rapidly within the next 3–5 years and will become a tool of choice for dynamic monitoring of patients on treatment or under active surveillance. In many instances, LB will also prompt further imaging workup and/or re-biopsy of tissue lesions. A frequently cited advantage is that obtaining LB fluids is much less invasive than SB or even imaging studies [19]. Scientific studies have increasingly provided evidence of the utility of LB for early diagnosis. ctDNA has been detected in up to 75% of pancreatic, ovarian, colorectal, bladder, breast, neck, hepatocellular, and gastroesophageal cancers and melanomas and in up to 50% of primary CNS, renal, prostatic, and thyroid cancers [17]. It has also been associated with metastatic burden in patients with non-small cell lung cancer (NSCLC) and small cell lung cancer (SCLC), among other tumors [19].

LB has thus, a wide potential of clinical applications and affords physicians a new tool for clinical management of difficult to treat patients with advanced stage cancers, prediction of treatment response, detection of recurrence, and traceability of tumor genome evolution over time [20, 21]. Studies demonstrate that ctDNA can be used in the routine management of lung cancer to monitor clonal evolution and identify treatment resistance [19, 22], particularly in patients with NSCLC who are treated with specific tyrosine kinase inhibitors (TKIs, such as gefnitinib, erlotinib, crizotinib, and ceritinib) [19]. Nearly half of NSCLC patients acquire resistance to TKIs and present *EGFR* T790M mutations; consequently, a second biopsy is required. Indeed, the European Society for Medical Oncology (ESMO) suggested LB as an alternative to tissue re-biopsy and presented LB as a validated method for monitoring progression of EGFR mutated patients [23, 24].

**Figure 3:**
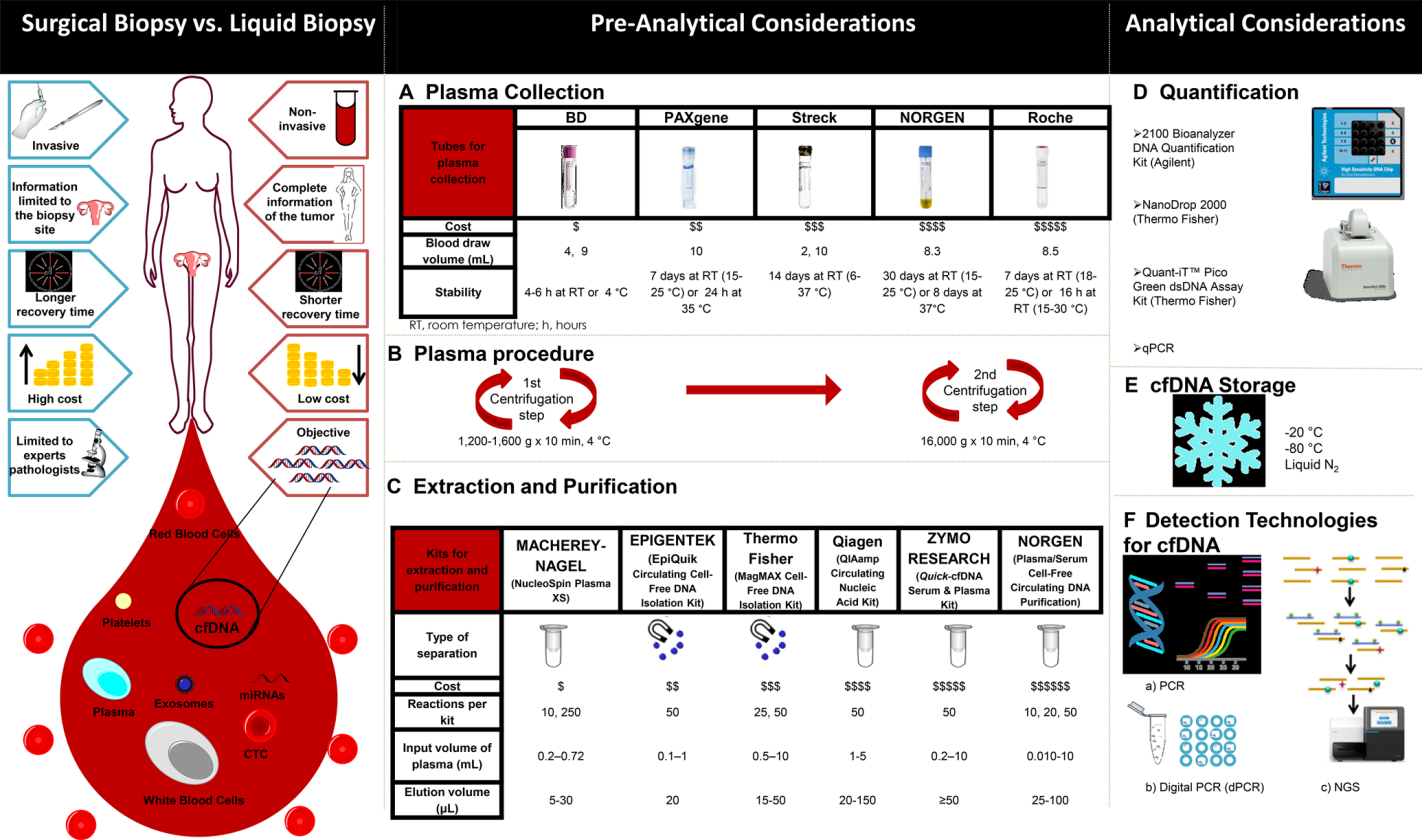
Comparison of features between surgical (tissue-based) and liquid biopsies (left) and overview of the various elements of the liquid biopsy workflow (**A**-**F**, right).

Figure 3 contrasts some of the advantages of LB relative to SB. LB can meaningfully augment SB by potentially sampling tumor heterogeneity more comprehensively and by revealing the dynamics of molecular changes of cancer cells while the patient is undergoing treatment [21].

LB has thus a wide potential of clinical applications and affords physicians with a new tool for clinical management of difficult to treat patients with advanced stage cancers, for prediction of response to treatment, for detection of recurrence, and for traceability of tumor genome evolution over time [20, 21].

### Circulating tumor DNA (ctDNA)

DNA is continuously released in fragments into the circulation through processes such as apoptosis and necrosis by both normal and cancerous cells [21, 25–27]. When released irrespective of cell of origin, it is typically referred to as cfDNA (cell-free DNA); but when released specifically by cancer cells, it is mostly referred to as ctDNA (circulating tumor DNA). Among the molecular characteristics of ctDNA are that it may harbor mutations, CNVs, methylation changes, or integrated viral sequences associated with the tumor [28–32]. ctDNA is mainly found in plasma and serum; however, it can also be isolated from ascites, breast milk, lymphatic and peritoneal fluids, bone marrow aspirates, urine, prostatic fluid, peritoneal lavage, sputum, cerebrospinal fluid, gastric juice, and biliary and even stool samples [33]. Circulating nucleic acids are removed from the blood by the liver and kidney and have variable circulating half-lifes ranging from 15 minutes to several hours [27, 34]. The concentrations of this genetic material in patients with cancer range from 0 to 1,000 ng/mL of blood, with an average of 180 ng/mL. In contrast, cfDNA in healthy subjects ranges from 0 to 100 ng/mL of blood, with an average of 30 ng/mL [27]. As a reference, a patient with a tumor burden of 100 g released 3.3% of ctDNA into the circulation [35].

In colorectal cancer, it has been found that ctDNA is more sensitive than the detection of serum protein biomarkers such as carcinoembryonic antigen (CEA), showing tighter changes in response to tumor resection and a greater predictive capacity for recurrence. In 2014, Bettegowda *et al*. [36] detected mutations in the *KRAS* gene of plasma ctDNA in a group of 206 patients with colorectal cancer, with a sensitivity of 87.2% and a specificity of 99.2%.

### Other source of LB-based biomarkers

#### Circulating tumor cells (CTCs)

CTCs have been discovered for Asworth in 1869 during an autopsy of a patient who had metastatic cancer [37]. They are cancer cells that detach from a primary or metastatic tumor site and are present in the circulation. Clinical evidence indicates that patients with metastases have 1–10 CTCs per mL of blood and they are rarely found in clinically healthy people or in people with non-malignant tumors. CTCs have been detected in different types of cancers, such as breast, ovarian, prostate, lung, colorectal, hepatocellular, pancreatic, head and neck, bladder, and melanoma [38]. There are commercial systems for their detection and isolation, of which the most used is the CellSearch^®^ system, an automated detection system for CTCs that uses anti-EpCAM antibodies, anti-CK antibodies, and anti-CD45 antibodies. CTCs are associated with a poor prognosis and are predictive of shorter progression-free survival and overall survival in patients treated with metastatic breast, colorectal, or prostate cancers who have CTC counts of ≥ 5, ≥ 3, or ≥ 5, respectively, per 7.5 mL of blood at any time during the course of the disease [39–41]. However, CTCs are often not detectable in patients with dysplastic or early malignant lesions, thus limiting their utility for early diagnosis or surveillance [42]. Besides their detection and isolation, CTCs can be cultured *in vitro* and to expand *ex vivo* for further analyses [43–46].

#### Exosomes

Exosomes are small round vesicles, 30–120 nm in diameter, and of endosomal origin carrying RNA, miRNAs, DNA, and proteins that are released by multiple cell types (including tumor cells) into the extracellular environment. Exosomes may mediate some form of communication between cells, being internalized by other cells [42, 47, 48]. They are found in biological fluids like blood, urine, saliva, pleural effusions, amniotic fluid, nasal secretions, bronchoalveolar lavages, cerebrospinal fluid, breast milk, and ascites [49–51]. Special features of exosomes have been associated with several types of cancer, such as pancreatic [52, 53], breast [54], gastric [55] colon [56], and ovarian [57].

#### miRNAs

MicroRNAs or miRNAs are small molecules of non-coding RNA, between 19 and 24 nucleotides in length, that act as regulatory molecules of gene expression, exerting function by hybridizing to inhibit the translation of mRNAs of its target genes [58–60]. Differential expression of miRNAs in patients with cancer has been described. miRNAs like those of the Let-7 family have been associated with lung cancer [61, 62]; miR15a/miR16a with chronic lymphocyte leukemia [63]; miR-34 family to neuroblastoma and colon cancer [64, 65]; miR-17–92 cluster with B-cell lymphoma, breast, colon, lung, stomach, prostate, and pancreatic cancers [66–68]; miR-21 with hepatocellular and breast cancer [69, 70]; miR-155 with diffuse large B-cell lymphoma and colorectal cancer [71, 72]; miR372/miR373 with testicular germ cell tumor [73]; and the miR-200 family with ovarian cancer [74]. In 2008, Mitchell *et al.* [75] showed that miRNAs may be ideal blood-based cancer biomarkers for three main reasons: 1) Expression of miRNAs is found frequently deregulated in cancer; 2) the expression patterns of miRNAs in cancer appear to be specific; and 3) miRNAs are usually highly stable in tissues fixed with formalin and, possibly, also in plasma or serum.

Based on this evidence as a whole, the expression levels of individual miRNAs and miRNA signatures are now linked to classification and prognosis of several human cancers.

### LB technology in the market

Several companies have been adapting genetic variation identification panels to include LB-based ctDNA as an input option. Boreal Genomics [76], Trovagene [77], RainDance Technologies [78, 79], Inivata [80], and Pathway Genomics [81] are examples of companies working on this technology (Table 1). Another example is Illumina's spin-off Grail that started in 2016 with the aim of developing an LB-based pan-cancer “molecular stethoscope.”

**Table 1:** Examples of LB panels in the market

Name of Panel	Company	Genes Analyzed	Reference
OnTarget	Boreal Genomics	Can include up to 100 mutations across multiple genes *(not listed)*	76
Trovera	Trovagene	*EGFR, KRAS*, and *BRAF*	77
ThunderBolts Cancer Panel	RainDance Technologies	*ABL1, EGFR, GNAQ, KRAS, PTPN11, RB1, MET, GNAS, ERBB2, AKT1, ALK, ERBB4, HNF1A, MLH1, RET, APC, EXH2, HRAS, MPL, SMAD4, ATM, FBXW7, IDH1, NOTCH1, SMARCB1, SMO, NPM1, IDH2, FGFR1, BRAF, CDH1, FGFR2, JAK2, NRAS, SRC, SKT11, PDGFRA, JAK3, FGFR3, CDKN2A, CSF1R, FLT3, KDR, PIK3CA, TP53, VHL, PTEN, KIT, GNA11,* and *CTNNB1*	78
ThunderBolts Myeloid Panel	RainDance Technologies	*ASXL1, BCOR, BCOR1, BRAF, CALR, CBL, CBLB, GATA1, FLT3, EZH2, ETV6, DNMT3A, CSF3R, CEBPA, GATA2, GNAS, HRAS, IDH1, IDH2, JAK1, JAK2, JAK3, KDM6A, KIT, KMT2A/MLL-PTD, KRAS, MEK1, MPL, PTEN, PML, PHF6, NRAS, NPM1, NOTCH1, MYD88, PTPN11, RAD21, RUNX1, SETBP1, SF3B1, SMC1A, MSC3, ZRSR2, WT1, U2AF1, TP53, TET2, STAG2,* and *SRSF2*	79
InVision	Inviata	*AKT1, ALK, BRAF, CCND1, CDKN2A, CTNNB1, EGFR, ERBB2, ESR1, FGFR1, FGFR2, FGFG3, GATA3, GNA11, GNAQ, GNAS, HRAS, IDH1, IDH2, KIT, KRAS, MAP2K1, MET, MYC, NFE2L2, NRAS, NTRK1, PDGFRA, PIK3CA, PPP2R1A, PTEN, STK11, TP53,* and *U2AF1*	80
CancerIntercept Detect/Monitor	Pathway Genomics	*BRAF, CTNNB1, EGFR, FOXL2, GNAS, KRAS, NRAS, PIK3CA,* and *TP53*	81

### Pre-analytical considerations of LB

In 2013, Messaoudi *et al*. [82] indicated that one of the main obstacles for the use of cfDNA in clinical practice is the heterogeneity of the various protocols for the manipulation and analysis of cfDNA. There are complex pre-analytical and analytical considerations to be taken into account when planning cfDNA analyses. Such considerations range from sample collection to interpretation of findings.

Among pre-analytical variables, the blood collection tube used is an important consideration, as it needs to provide the necessary conditions for the stability of the cfDNA [83]. There are several collection tubes on the market as illustrated in Figure 3A. One of the applications in which this is especially critical, because of the need for long-term stable cfDNA, is in studies (such as multicenter clinical trials) in which blood samples are sent for analysis to a laboratory in another region.

Plasma processing is another pre-analytical factor to consider. Messaoudi and colleagues report that performing a double centrifugation during plasma isolation is ideal for minimizing contaminating nuclear DNA from white cells that otherwise would contaminate and dilute true cfDNA [82]. Double centrifugation has thus become the current gold standard for prospective plasma biobanking (Figure 3B).

The choice of procedure for the extraction and purification of cfDNA is, however, the most important pre-analytical factor to be taken into account. For this, a wide variety of kits exist that allow isolating cfDNA from several sources, particularly from plasma. In Figure 3C, some of the popular commercially available kits for the extraction and purification of cfDNA, based on the separation by silica columns or by magnetic beads, are listed. Once the genetic material is isolated, it is necessary to perform an analysis to confirm the quality of the sample. For this, the companies producing the extraction and purification kits offer various quality control methodologies as indicated in Figure 3D. The choice of reagents and equipment to be used will depend to a great extent on availability, accessibility, budget, analytical sensitivity, and final readout desired (such as end-point PCR, real-time PCR, digital PCR (dPCR), microarrays, or NGS).

Finally, storage and freezing conditions of ctDNA need to be considered (Figure 3E). Our own data shows that storage at –20 °C, –80 °C, and in vapor phase liquid nitrogen (below –150 °C) are equivalent once the DNA has been extracted. Long-term stability of cfDNA in plasma and serum that is frozen prior to extraction remains to be studied.

### Analytical possibilities of LB

The methods for ctDNA analysis can be divided into point mutations analysis; detection of somatic mutations as biomarkers and whole genome analysis (WGA); and detection of rearrangements and chromosomal copy-number changes (Table 2; Figure 3F) [84].

**Table 2:** Comparison of methodologies for ctDNA analysis

Method	Technology	Sensitivity	Type of Alteration
qPCR	ARMS-Scorpions PCR	0.05–0.1%	Known point mutation
	Clamping PCR	0.1–1%	
	TaqMan	0.1–1%	
Digital PCR	Beaming	0.01%	
	ddPCR	0.001%	
Target sequencing	TAm-Seq	>2%	Point mutations in gene regions; structural alterations in gene regions
	SAFE-SeqS	0.1%	
	CAPP-Seq	0.01%	
Whole genome sequencing	Digital karyotyping	0.001%	Genome-wide copy-number changes; personalized
	PARE	0.001%	genome-wide rearrangements

ARMS, amplification refractory mutation system; BEAMing, beads, emulsion, amplification, magnetics; CAPP-Seq, cancer personalized profiling by deep sequencing; ddPCR, droplet digital PCR; PARE, parallel analysis of RNA ends; qPCR, quantitative PCR; SAFE-SeqS, safe-sequencing system; TAm-Seq, tagged-amplicon deep sequencing.

In point mutations analysis, qPCR, dPCR, and targeted sequencing can be included; these technologies are highly sensitive (< 1%) and allow detection of a low tumor fraction in plasma DNA, while WGA can be used in high tumor fraction situations (sensitivity > 10%) [17]. In this sense, the best options for diagnosis are methodologies based on PCR since they allow detection of low levels of ctDNA. NGS methodologies are increasing the sensitivity and throughput necessary for diagnostic ctDNA analysis [85].

### Potential limitations of LB

While LB is doubtlessly an extremely powerful addition to the diagnostic tool set, the approach also suffers from several technology-inherent limitations that need to be discussed and considered.

First, LB may have lower sensitivity than SB for rare variants. This is because LB attempts detection of alterations in peripheral fluids rather than the tumor itself (volume dilution) and in a background of non-altered cfDNA from cellular sources other than the tumor (for example, in a patient with other co-morbidities such as sepsis, abundant cfDNA may be circulating that is derived from non-cancerous cell compartments). Second, while LB may be able to pick up heterogeneity (via ultra-deep NGS of ctDNA), tracing tumor heterogeneity back to multiple simultaneous lesions (e.g., primary vs. multiple metastatic sites) and pinpointing which clones dominate which site may be close to impossible without combining LB with smartly targeted SB procedures. In addition, it is plausible that various distinct lesions in a patient would each shed variable amounts of ctDNA and thus LB would not just be a volume-proportional mixture of contributing lesions (thus making the heterogeneity problem even harder to untangle). Some lesions, e.g., brain metastases, may shed little to no ctDNA into the circulation. Finally, LB could potentially pick up physiologically continuously occurring but clinically inconsequential mutational events in other high turnover compartments, such as the bone marrow. This would further complicate the interpretation of rare alleles detected by ultra-deep sequencing of ctDNA.

### Evolutional analysis of cancer by ctDNA

Besides the benefits mentioned previously, LB may also allow for evolutional analysis of cancer in real time. The subclonal dynamics of ctDNA has been characterized in several studies, which demonstrate that ctDNA can be used to detect emergence of resistance to treatment [86–88]. LB has allowed to carry out studies on frequency, identity, and evolution of subclonal genetic alterations that had previously been very limited due to the difficulty of serially accessing tumor tissue [88, 89]. Abbosh *et. al.* performed a a phylogenetic subclone analysis in NSCLC employing ctDNA over a period of 231 days and identified single nucleotide variations (SNV) that had not been identified in the primary tumor, suggesting that ctDNA can be used when subclones of the primary tumor are found in low quantities. In addition they analyzed patients who had liver metastases in which subclones were identified originating in the primary pulmonary tumor [88]. Furthermore, they were able to associate the ctDNA with the histological type of the tumor. Imamura *et. al.* determined that ctDNA is a specific tumor marker for assessing the response to treatment and the molecular dynamics of NSCLC-related oncogenes [90].

### Summary

LB is at the dawn of a new era of cancer “theranostics”, being a non-invasive addition to SB. LB is capable of generating valuable information about cancer almost in real time that can be used to reveal the genetic features of individual tumors, thus improving early detection, prognostication, and monitoring treatment responses and eventual resistance. We have reviewed the current state of the art of LB-related analytics and provide an outlook of its clinical utility.
